# The Scarce Drugs Allocation Indicators in Iran: A Fuzzy Delphi Method Based Consensus

**DOI:** 10.22037/ijpr.2019.1100678

**Published:** 2019

**Authors:** Leila Zarei, Farzad Peiravian, Mir Saman Pishvaee, Bahar Aghababaei

**Affiliations:** a *School of Pharmacy, Shahid Beheshti University of Medical Sciences, Tehran, Iran. *; b *Health Policy Research Center, Shiraz, Iran. *; c *School of Industrial Engineering, Iran University of Sciences and Technology, Tehran, Iran.*

**Keywords:** Resource Allocation, Delphi Techniques, Health-care Resources, Scarce Drugs Allocation, Needs-Based Resource Allocation, Pharmaceutical Policy, Fuzzy Theory

## Abstract

Almost all countries are affected by a variety of drug-supply problems and spend a considerable amount of time and resources to address shortages. The current study aims to reach a consensus on the scarce drug allocation measures to improve the allocation process of scarce drugs in Iran by a population needs-based approach. To achieve the objective, two phases were conducted. Firstly, a set of population-based indicators of health needs were identiﬁed by reviewing the literature and were scrutinized by fifty academics/executives who were specialists in pharmaceutical resource allocation. In the second phase, a structured process, based on the Delphi technique requirements, was performed to finalize the indicators. The yield of literature review step was about 20 indicators, which was based on availability of data in Iran, 16 indicators were added to the next step and formed the initial questionnaire. Based on the results of the first questionnaire, only 3 indicators were rejected and 13 indicators were added to the Delphi phase. Then, in Delphi phase, the consensus was built after three Rounds. In addition to the burden of endemic, special, rare, and incurable diseases, traumatic diseases and total population of each province were the main measures. Furthermore, total mortality rates and the number of pharmacies in each province were on the border; hence, the monitoring team made the decision about inclusion or exclusion of such indicators. Other measures were in the range of ′important′ ones. To reach a higher effective and efficient process of resource allocation, the paper suggests the use of a population needs-based approach in Iran′s pharmaceutical sector. The scarce drug allocation indicators extracted in this study can make a considerable contribution to preventing, controlling, and mitigating drug shortages.

## Introduction

The shortage related to drugs is an intricate and worldwide phenomenon that normally occurs in different parts of the world. This problem is an important subject for policy makers in each health-care system, because shortage in drug supplies might further lead to suspensions of treatments for patients and care interruptions (1, 2). Under the drug shortage circumstances, all related health-care stakeholders can be affected; hence, cooperation is highly required to reduce the drug shortages and to manage the condition (3).

During such shortage periods, the issue of reaching a higher value without increasing the amount of resources is raised. (4). On the one hand, the equity in health-care resource allocation is important and is considered as one of the highest influential subjects for different related groups such as politicians and researchers (5–8). On the other hand, more studies are required to uncover the association of this issue with inequalities in socioeconomic subjects (9). 

In Iran, resource allocation systems tend to allocate resources on the basis of past levels of service utilization. Under these systems, there are no specific indicators for optimal allocation, and any existing inequities in access to care will continue (10). In case of shortage, Iran Food and Drug Administration (IFDA) has implemented a distribution policy at provincial level, the considered indicators at this time are the population of each province, the number of pharmacies, and the number of practitioners in each province. But these criteria fail to thoroughly meet the patients′ demands around the country (10). In this regard, a major challenge for health-care policy makers in Iran is to identify and implement the main indicators of needs-based resource allocation to Iranian provinces which are consistent with the objective of Iranian health-care policy and national drug policy (NDP) that emphasizes on equity in access (11).

Studies in Iran are mainly focused on the geographical distribution of resources in different population levels without taking the papulation requirements into consideration (5, 12). Utilization of needs-based allocation of health-care resources was recommended to policymakers by some studies in Iran (5, 10, 12, 13). To the best of our knowledge, previous attempts at resource allocation of health-care system provide no detailed attention to the scarce drugs. In addition, the needs-based resource allocation is a missing link in this area. Since the adoption of the most effective indicators of needs for scarce drug allocation plays a pivotal role in preventing and controlling drug shortage, identification and consensus the valid indicators by a fuzzy Delphi study seems most appropriate in accordance with the objectives of this study.

*Theoretical Framework*

In this section, a brief review of theoretical framework, including the needs-based approach and fuzzy Delphi method, is presented.

*Needs- based Approach*

Health-care systems apply various methods of allocating resources to sub-geographic areas and groups. The four commonly used methods are: 1) political patronage 2) historical allocations 3) bids by local governments and 4) and needs-based resource allocation formulae (14).

The most reliable resource allocation framework related to the highest requirements of the population, known as needs-based approach, leads to the allocation of health-care resources through the characteristics of the population so that the use of inadequate health-care supplies can be optimized (15). In this approach, the amount of each population’s allocation was determined independently from the existing levels of utilization by that population; rather, it is related to the characteristics of each population in the context of the corresponding characteristics of the entire population (e.g. provincial versus national) (16).

Needs-based resource allocation has globally been an issue (17). A number of countries in Europe (Germany, Switzerland, UK, Sweden, and Spain) have adopted scientific needs-based allocation models (15, 18). According to Gugushvilli (19), the United Kingdom′s National Health Service (NHS) was a pioneer in the field of needs-based resource allocation in 1977. While the above-mentioned countries exploited this approach to address the issue of inequity, a proper and applicable set of procedures and measures were highly required to guide resource allocation in most countries with low or medium earnings (15). 

Health-care needs can be measured directly or indirectly; direct measures are based on the quantifications of the precise healthcare services required to improve the health status of an individual or a group is based on the assessment of health-care professionals. Alternatively, the need for health-care can be estimated through indirect measures such as mortality, morbidity, socio-demographic characteristics, and socio-economic characteristics or measures of deprivation (16, 20). According to McIntyre and Anselmi (2012), the most common indicators of needs are: demographical compositions, socio-economic status, ill-health levels, and the size of the population (10, 22). Clearly, with the lack of any gold standard measures of population needs, it is important to choose a valid, reliable, and responsive indicator of (or proxies for) health-care needs (10, 22).

*Fuzzy Delphi Method*

In the real world, decision-makers encounter complex, and sometimes, multi-dimensional problems. Therefore, in many cases, the problems related to decision making are not clearly deﬁned; as a result, quite a few problems in the real world are not random but are intrinsically fuzzy; subsequently, the application of probability in numerous cases has not been satisfactory enough (23). Alternatively, the use of fuzzy theory in problems related to decision making has revealed proper practical results (24). The main characteristic of the fuzzy theory in this area provides a higher ﬂexible framework (25). Accordingly, even if the unavailability or expensiveness of the accurate information or in the situation where the subjective inputs are required for model evaluation, the application of the fuzzy Delphi is valid (26). As a flexible method, Delphi is built and manufactured on the following basic concepts: “structured questioning, iteration, controlled feedback, and anonymity of responses”(27). 

Most Delphi users try to employ the policy formulation or decision making (26); however, Delphi is considered as a tool for analyzing the policy issues (26). Practically, the consensus-oriented Delphi was utilized in numerous fields such as analyses of resource allocation, technological forecasting, strategic planning, formulation of the policy, and technology assessing (26). A currently applicable case of Delphi can be used in health-care study areas, which currently has a number of Delphi practitioners (17, 27). This technique could be included in the groups of methods applied for indicator development (28).

*Methods*

**Figure 1:**
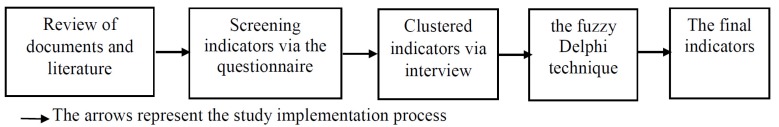
The graphical presentation of the study implementation process

In the process of implementing the study, as shown in Figure1, firstly, a committee of three experts and advisers in drug policy field, analysis and planning, and also in Delphi technique - have forged the monitor team, and they continuously provided assistance in different aspects of all parts in the study.

To achieve the study objective, two phases were conducted in the study. In the ﬁrst phase, a set of population-based measures of health needs were identiﬁed based on a wide literature review. Then, the measures obtained from the previous step formed the initial questionnaire and were scrutinized by fifty academics and executives who were specialists in pharmaceutical resource allocation and distribution. Thus, over 60 potential respondents at the national level, 50 people (e.g., Vice-chancellors for food and drug in universities of medical sciences and other experts in the field), who were directly impacted by the consequences of drug shortage, screened the indicators. In order to evaluate the results of the first questionnaire, Content Validity Ratio (CVR) was applied. Content validity is mandatory for the topic-related areas (29). Lawshe (1975) developed a proper method for measuring the content validity (30), and he suggested that each Subject Matter Expert (SMEs) raters on the judging panel should respond to questions of “Is the measure ′a) essential,′ ′b) useful, but not essential,′ or ′c) not necessary′ to the construct performance?” If a higher number of individuals participating in the test agree with a particular item or with the measure ‘is essential’, higher levels of content validity would exist. Lawshe provided a table of critical values for the CVR which is known as Schipper′s table. Wilson, Pan, and Schumsky (31) later modified the table. The final decision to accept or reject items is based on this table. Consequently, in this phase, the population-based measures were sifted and clustered in accordance with national drug policy and their applicability in Iran. The second phase was based on the fuzzy Delphi technique. Based on the composition of the members and the homogeneity of the target group, Delphi can be used by six to fifty participants (32, 33); in the fuzzy Delphi phase, it was anticipated that nearly 15 participants would be included in the study, but, finally, the phase was held by 9 individuals, due to the lack of participation. The group of respondents represented some of the highest authorities in the field. Such a group comprised the cognizant IFDA Deputy, prominent politicians, and researchers in the resource allocation field. It must be noted that they participated voluntarily and freely.

Even though several forms of fuzzy numbers are available, the trapezoidal and triangular forms are frequently used to represent fuzzy numbers. In this study, trapezoidal fuzzy numbers were applied. In fuzzy multiple criteria decision-making problems, various trapezoidal fuzzy numbers can orderly be ranked by means of portrayal of their curves. On the condition that its order cannot be ranked by the afore-mentioned method, other approaches could be applied instead. In the current study, the procedure proposed by Cheng was employed (23). 

## Results

Several mechanisms, which are classified as supply and demand side mechanisms, can be used for the allocation of scarce health-care resources. The indicators in each side are different, and different countries used a simple, or a single indicator or composite indicators to weigh district population. In many countries where the concept of needs has been incorporated in resource allocation, composite indicators of socioeconomic status such as deprivation and asset indices have been utilized. In developing countries, the application of demographic and socioeconomic status such as size of population, age, sex, and health indices are common. The yield of literature review was about 20 indicators, which could have been potentially applied in Iran, but due to the situation, limited information, as well as the inaccessibility of reliable information in Iran, employing the above-mentioned indicators seemed illogical. Therefore, 16 indicators were transferred to the next step and formed the initial questionnaire. By evaluating critical values of CVR, 3 indicators were rejected, for their CVR scores were below 0.24; furthermore, some new indicators were introduced by this study. This means that according to the comments of participants in the phase, some measures were changed as follows: Population and mortality rates adjusted by age and gender were changed to total population and total mortality; the number of hospital beds was changed to total inpatient bed occupancy rate; the pharmaceutical quotas in the past was omitted, but the burden of traumatic diseases was added. Then, based on the results of this phase, the Delphi questionnaire was designed. 

In the following, based on the Delphi technique requirements, a structured process was performed to reach a consensus in order to improve the allocation of scarce drugs in Iran. Then, the experts’ opinions were fully reviewed by using a series of ‘Rounds’. Rounds were held until group consensus was reached based on Cheng′s proposed method (23). The following summarizes the computational process: 

At first, the linguistic variables were used by decision-makers to evaluate the significance of the measures to improve the allocation of scarce drugs in drug shortage crises in Iran. Table 1 indicates the transfer of the linguistic variables to trapezoidal fuzzy numbers.

The monitoring team provided a significant amount of time for participants to carefully fill out the questionnaires. 

The results of the first questionnaire (Round1), including both the number of selected linguistic variables and the fuzzy average of experts′ answers are provided in Table 2. This calls for calculating the mean related to all trapezoidal fuzzy numbers by formula 1 and 2. 

*Ã* ^(i)^ *= *(α_1_^(i) ^.α_2_^(i)^.α_3_^(i)^.α_4_^(i)^) , i=1,...,n.                    (1)

$\overset{\text{\textasciitilde{}}}{A_{m}}=\left(a_{\mathrm{m1}}.a_{\mathrm{m2}}.a_{\mathrm{m3}}.a_{\mathrm{m4}}\right)=\left(\frac{1}{n}\sum_{i=1}^{n}a_{1}^{\left(i\right)}.\frac{1}{n}\sum_{i=1}^{n}a_{2}^{\left(i\right)}.\frac{1}{n}\sum_{i=1}^{n}a_{3}^{\left(i\right)}.\frac{1}{n}\sum_{i=1}^{n}a_{4}^{\left(i\right)}\right)$

(2)

Where:

*Ã* is a trapezoidal fuzzy number; i represents Experts; a_1_, a_2_, a_3_ ,and a_4_ are Ã membership; *Ã*_m _is the average (mean) of all *Ã*^(i)^; a_m1_, a_m2_, a_m3_, and a_m4_ are the average of all a_1_^(i)^, a_2_^(i)^, a_3_^(i)^, and a_4_^(i)^ , n = 4.

Then, based on the results of the first round, the differences between the answer of each expert to average of the experts’ answers were computed by formula 3. 

$\left(a_{\mathrm{m1}}-a_{1}^{\left(i\right)}.a_{\mathrm{m2}}-a_{2}^{\left(i\right)}.a_{\mathrm{m3}}-a_{3}^{\left(i\right)}.a_{\mathrm{m4}}-a_{4}^{\left(i\right)}\right)=\left(\frac{1}{n}\sum_{i=1}^{n}a_{1}^{\left(i\right)}-a_{1}^{\left(i\right)}.\frac{1}{n}\sum_{i=1}^{n}a_{2}^{\left(i\right)}-a_{2}^{\left(i\right)}.\frac{1}{n}\sum_{i=1}^{n}a_{3}^{\left(i\right)}-a_{3}^{\left(i\right)}.\frac{1}{n}\sum_{i=1}^{n}a_{4}^{\left(i\right)}-a_{4}^{\left(i\right)}\right)$

 (3)

Where:

*Ã* is a trapezoidal fuzzy number; i represents Experts; a_1_, a_2_, a_3_ ,and a_4_ are *Ã* membership; 

*Ã*_m_ is the average (mean) of all *Ã*^(i)^; a_m1_, a_m2_, a_m3_ ,and a_m4_ are the average of all a_1_^(i)^, a_2_^(i)^, a_3_^(i)^, and a_4_^(i)^ , 

n = 4.

Accordingly, preparation of a summary of the first Round results began shortly after the first completed Round. In the second Round, the second questionnaires along with an abstract of the results in first Round were given to the participants. They were allowed to revise their personal opinions according to the opinions of others.

Again the number of selected linguistic variables and the fuzzy average of the experts′ answers were computed by formulae 1 and 2. The results of the second questionnaire (Round 2) are shown in Table 2.

The method of Delphi is based upon the consensus achieved by panelists. In order to investigate the achieved consensus, the difference between the means of two implemented rounds was compared; and accordingly, the decision is made whether to continue or stop the Delphi process. 

The mean of two rounds was calculated by formula 4 and are shown in Table 3. If the difference was lower than a specified threshold (d_i_≤ 0.2) the consensus was made and the process was stopped.

(4) $$S\left(A_{\mathrm{m2.}}A_{\mathrm{m1}}\right)=\left|1/4[\left(a_{\mathrm{m21}}+a_{\mathrm{m22}}+a_{\mathrm{m23}}+a_{\mathrm{m24}}\right)-\left(a_{\mathrm{m11}}+a_{\mathrm{m12}}+a_{\mathrm{m13}}+a_{\mathrm{m14}}\right)]\right|$$

As Table 3 displays, by doing the second round, some measures failed to reach the specified threshold (d_i_≤0.2). Therefore, after identifying the differences between opinions by formula 4, the third round was implemented, as was in the previous rounds. The results are shown in Table 4.

Next, in order to check the achieved consensus, the difference between the means of round 2 and round 3 was examined; the results are provided in Table 3. Since all the requirements of reaching the consensus were met, the process was stopped.

Finally, based on Table 4, the measures that were in the range of ′very unimportant to neutral′ (the average of the third-round was below 6) had to be eliminated. Thus, the deprivation index of the province and population growth rate was eliminated. In addition to the burden of endemic, special, rare and incurable diseases, traumatic diseases and total population of each province were the main measures because they were in the range of ′very important.′ Furthermore, the population of non-resident patients, total inpatient bed occupancy rate, number of prescriptions, the number of GPs and specialists in each province were in the range of ′important′ measures. Other measures, including total mortality rates and the number of pharmacies were on the borderline. Since mortality rates are hidden in burden of disease measures, and the number of prescriptions can act as a proxy of the number of pharmacies, the monitoring team decided not to include these measures in potential allocation model. The number of prescriptions was used because it can show the final medicine demand better than the number of pharmacies. In other words, the number of prescriptions can indicate the real demand more accurately.

## Discussion

Since shortages related to the drugs are frequent in health-care systems and stem from a wide variety of reasons, the future drug shortages are unknown and unpredictable. Hence, numerous organizations and stakeholders have been endeavoring to manage current shortages and to improve evidence-based decision making for future shortages. The primary aims of this study were to define and determine the needs-based measures of scarce drug allocation in the health-care system of Iran by utilizing Delphi technique to improve the allocation of these valuable resources. The results of this study pave the way for policy makers to improve their decisions concerning resource allocation, and these population needs-based measures could be included in the allocation formula of scarce drugs as well.

Among the 13 reviewed measures, eight ones such as the burden of endemic, special, rare and incurable disease, and traumatic diseases, total population, the population of non-resident patients, total inpatient bed occupancy rate, the number of prescriptions and the number of GPs and specialists were approved. Our findings revealed that eight of the common measures/indicators of resource allocation enjoyed face validity to measure needs in Iran and are also currently used in needs-based resource allocation formula in other countries (15). In fact, the needs-based resource allocation indicators in different countries are conceptually similar and these indicators as discussed above (population, deprivation and health needs indicators) are a significant role in resource allocation of many countries. In the Southern African regions, England, Namibia, and Spain, the size of population has been utilized in the health-care resource allocation (18). The burden of diseases is a popular indicator in countries like southern African countries, the US, Scotland, Canada, and Wales (34). Although the level of deprivation of each province is another popular indicator in health-care scarce resource allocation (e.g. in Australia), it was not approved by our results. The reason is most probably related to the concern about the accessibility of data. Other indicators are common in composite indicator of health index and based on the results of current study, their application is justifiable (15, 21).

**Table 1:** Linguistic variables

**Very unimportant**	(0,0,0.5,2.5)
**Unimportant**	(0,2,3,5)
**Neutral**	(2.5,4.5,5.5,7.5)
**Important**	(5,7,8,10)
**Very important**	(7.5,9.5,10,10)

**Table 2:** The results of the first and second Round

**Second Round**		**First Round**		**Measures/ Indicators**
**Fuzzy average**	**Linguistic variables** *	**Fuzzy average**	**Linguistic variables** *	
(6.4, 8.4, 9.1, 9.72)	(6,2,1,0,0)	(6.1, 8.1, 8.8, 9.72)	(5,3,1,0,0)	Total population of each province
(4.4, 6.4, 7.4, 9.44)	(0,8,0,1,0)	(5, 7, 7.9, 9.44)	(2,6,0,1,0)	The population of non-resident patients in each province
(1.9, 3.9, 4.9, 6.94)	(0,3,1,5,0)	(1.9, 3.9, 4.9, 6.94)	(0,3,1,5,0)	Population growth rate
(3.6, 5.6, 6.6, 8.61)	(0,6,1,2,0)	(2.8, 4.8, 5.8, 7.75)	(0,4,2,3,0)	Total mortality rates of each province
(5.8, 7.8, 8.6, 9.44)	(5,3,0,1,0)	(5.6, 7.6, 8.3, 9.44)	(4,4,0,1,0)	Total inpatient bed occupancy rate of each province
(5.6, 7.6, 8.3, 9.44)	(4,4,0,1,0)	(5, 7, 7.8, 9.17)	(3,4,1,1,0)	Number of prescriptions
(3.6, 5.4, 6.3, 8.06)	(1,5,0,2,1)	(3.6, 5.4, 6.3, 8.06)	(1,5,0,2,1)	The number of GP in each province
(6.1, 8.1, 8.9, 10)	(4,5,0,0,0)	(5.8, 7.8, 8.6, 9.72)	(4,4,1,0,0)	The number of specialist doctors in each province
(4.4, 6.4, 7.4, 9.17)	(1,6,1,1,0)	(4.2, 5.9, 6.8, 8.33)	(2,4,1,1,1)	Number of pharmacies in each province (independent pharmacies and pharmacies of health centers)
(2.5, 4.3, 5.2, 6.94)	(1,3,0,4,1)	(3.1, 4.8, 5.7, 7.5)	(1,4,0,3,1)	Deprivation index of the province
(7.5, 9.5, 10, 10)	(9,0,0,0,0)	(7.2, 9.2, 9.8, 10)	(8,1,0,0,0)	The burden of endemic diseases in each province
(7.5, 9.5, 10, 10)	(9, 0,0,0,0)	(7.2, 9.2, 9.8, 10)	(8,1,0,0,0)	The burden of special, rare and incurable diseases** in each province
(7.5, 9.5, 10, 10)	(9,0,0,0,0)	(6.9, 8.9, 9.6, 10)	(7,2,0,0,0)	The burden of traumatic diseases in each province

* From Very important to Very unimportant, respectively.

*** Exmp: hemophilia, thalassemia , diseases requiring dialysis, cancers, multiple sclerosis, metabolic disorders and diabetes.

**Table 3:** The difference between the mean of two implemented Rounds (Round 1 and 2; Round 2 and 3)

**Difference ** ***(Round 2 and 3)***	**Difference ** ***(Round 1 and 2)***	**Measures**
0.19	0.194	Total population of each province
0.28	-0.39	The population of non-resident patients in each province
0.83	0	Population growth rate
0.83	0.83	Total mortality rates of each province
0.39	0.19	Total inpatient bed occupancy rate of each province
-0.19	0.47	Number of prescriptions
0.56	0	The number of GP in each province
0	0.28	The number of specialist doctors in each province
-0.19	0.56	Number of pharmacies in each province (independent pharmacies and pharmacies of health centers)
-1.11	-0.56	Deprivation index of the province
0	0.19	The burden of endemic diseases in each province
0	0.19	The burden of special, rare and incurable diseases in each province
0	0.39	The burden of traumatic diseases in each province

**Table 4:** The results of the third Round

**Fuzzy average**	Linguistic variables*	**Measures**
(6.67, 8.67, 9.28, 9.72)	(7,1,1,0,0)	Total population of each province
(4.7, 6.7, 7.7, 9.72)	(0,8,1,0,0)	The population of non-resident patients in each province
(2.8, 4.8, 5.8, 7.78)	(0,5,0,4,0)	Population growth rate
(4.4, 6.4, 7.4, 9.44)	(0,8,0,1,0)	Total mortality rates of each province
(6.4, 8.4, 9, 9.44)	(7,1,0,1,0)	Total inpatient bed occupancy rate of each province
(5.3, 7.3, 8.1, 9.44)	(3,5,0,1,0)	Number of prescriptions
(4.2, 5.9, 6.8, 8.61)	(1,6,0,1,1)	The number of GP in each province
(6.1, 8.1, 8.9, 10)	(4,5,0,0,0)	The number of specialist doctors in each province
(4.2, 6.2, 7.2, 9.17)	(0,7,1,1,0)	Number of pharmacies in each province (independent pharmacies and pharmacies of health centers)
(1.1, 3.1, 4.1, 6.11)	(0,2,0,7,0)	Deprivation index of the province
(7.5, 9.5, 10, 10)	(9,0,0,0,0)	The burden of endemic diseases in each province
(7.5, 9.5, 10, 10)	(9,0,0,0,0)	The burden of special, rare and incurable diseases in each province
(7.5, 9.5, 10, 10)	(9,0,0,0,0)	The burden of traumatic diseases in each province

In shortage periods, the IFDA conducts a controlled allocation/distribution program to improve the equity and efficacy in Iran’s health-care system (8), and, at certain times, the shortages related to drug supplies in Iran are a type of health-care rationing. Like other countries, explicit health-care rationing in Iran is unpopular; however, implicit (and sometimes erratic) rationing can balance resources and maintain the system (35). It is proposed that some considerations should be taken to identify the determinants of variations among the people in each province for a rational rationing so that the limited resources can meet health-care needs (10). The final measures derived from this study, as the most important indicators of population′s needs, can play this role. 

As Gibson *et al*. (2012) demonstrated, definition and measurement of the health needs can minimize the possibility of local drug shortage occurrence (36). The findings of the current research are not only in line with Gibson′s recommendation but also confirm that a needs-based approach can effectively make a contribution to the IFDA in preventing and managing drug shortages. 

Regularly, drug shortages must be efficiently addressed with the efforts on redistribution of resources and conservation of supplies to restrict potential influences on patient-related care. Therefore, in this situation, certitude of effectively using limited health-care resources is essential, and it can improve an efficient reallocation of supplies to enhance health-care service equity (21). 

The results of the current study provide the prerequisites for calculations of the needs of each province; therefore, the above-mentioned goals can be achievable.

Briscombe, Sharma, and Saunders 2010 stated that the resource allocation to provinces must be in line with health needs of each provinces and the resource allocation formula will need to take into account different population sizes, disease burden, and other factors. In Kenya, their suggestion model was assessed for using in resource allocation (18). The findings of the current research support the model obtained in the previous study.

In line with the research of Kaltenthale *et al*. 2004, by a systematic review, the current study extracted population needs-based resource allocation indicators; as a result, single and composite indicators were identified (22). Like other studies, the results of this study confirmed that the population size in each geographic area, the burden of disease, demographic composition, and socio-economic status are the most common indicators of needs in the resource allocation methods (21). 

Updated population needs-based indicators were collected by a number of national surveys (population census). For example, population size of each province is a common indicator that is the basis for calculation of many other indicators as well. Then, any change in demographic policies, either directly or indirectly, can lead to a noticeable change in the final model. In contrast, the mortality rate can bring about deviations in the final model, meaning a significant portion of the pharmaceutical expenditure is spent on patients whose treatment does not culminate in death. On the other hand, the geographical distribution of drug needs is different from the geographical distribution of death in different parts of a given country. 

## Conclusion

Achieving high levels of efficiency and success in resource allocation procedures can be accessible provided that the resource distribution is commensurate with population requirements. 

Given the specific context of the Iranian health-care system, it is necessary to design a needs-based resource allocation model that is compatible with the health-care system features. This model can improve the equity of care and provide a practical guide for managers and policy makers to promote evidence-based decision making. It is suggested that the final measures of this study be divided to indicators related to the access, efficacy, and equity in line with national drug policy (NDP). Thus, it is recommended that future studies, based on our findings, should design a model for scarce drugs allocation for Iranian health-care system. 

On the other hand, the IFDA strategy for drug shortage prevention and mitigation has the potential to decrease the burden of drug shortages impacting the patients. Future revisions of the IFDA strategy should incorporate specific province considerations, and this study has separately addressed the difference of the needs of each province; as a result, it can make a distribution to meet their needs. 
